# Association Between Age, Race, Ethnicity, and Body Mass Index and Time to Endometriosis Diagnosis

**DOI:** 10.1097/og9.0000000000000072

**Published:** 2025-03-27

**Authors:** Marisa R. Imbroane, Yasamin Fazeli, Hanna Kim, Tommaso Falcone, Elliott G. Richards

**Affiliations:** School of Medicine, Case Western Reserve University, the Cleveland Clinic Lerner College of Medicine, and the Department of Reproductive Endocrinology and Infertility, Cleveland Clinic, Cleveland, Ohio.

## Abstract

**OBJECTIVE:**

To assess the associations of time to endometriosis diagnosis after presentation for pelvic pain between patients of different ages, races, ethnicities, and body mass index (BMI) ranges.

**METHODS:**

This retrospective study was conducted with the U.S. Collaborative Network on the TriNetX research network of health care organizations using International Classification of Diseases, Tenth Revision (ICD-10) codes. Race, ethnicity, and BMI cohorts consisted of 18- to 44-year-old female patients with diagnoses of endometriosis (ICD-10 N80) and pelvic–perineal pain (ICD-10 R10.2). Age cohorts consisted of female patients aged 18–30 years or 31–44 years, with the same two diagnoses. The BMI cohorts were categorized as lower than 19.9 (ICD-10 Z68.1), 20–29 (ICD-10 Z68.2), and higher than 30 (ICD-10 Z68.3, Z68.4). Patients were matched for race, ethnicity, and age at pelvic pain diagnosis. We assessed time to endometriosis diagnosis after an initial diagnosis of pelvic and perineal pain using Kaplan–Meier analysis and log-rank test with a value of *P*<.05 considered significant.

**RESULTS:**

For patients aged 18–30 years (n=13,662), the median time to endometriosis diagnosis was 0.49 years; for patients aged 31–44 years (n=13,662), the median time to diagnosis was 3.10 years (χ^2^=3,503, *P*<.001). In a comparison of Black and White patients (n=12,242 patients in each group), the median time to endometriosis diagnosis for Black patients was 1.34 years compared with 0.67 years for White patients (χ^2^=128, *P*<.001). For Asian (n=2,453) and White (n=2,453) patients, the median time to endometriosis diagnosis was 0.58 years for Asian patients and 0.64 years for White patients (χ^2^=0.68, *P*=.41). For Hispanic and non-Hispanic patients (n=10,640 patients in each group), the median time to endometriosis diagnosis was 1.11 years for Hispanic patients and 0.68 years for non-Hispanic patients (χ^2^=27, *P*<.001). For BMI lower than 19.9 (n=1,448) and BMI 20–29 (n=1,448), the median time to endometriosis diagnosis was 1.15 years for BMI lower than 19.9 and 1.35 years for BMI 20–29 (χ^2^=2.8, *P*=.10). For BMI higher than 30 (n=1,719) and BMI lower than 19.9 (n=1,719), the median time to endometriosis diagnosis was 1.52 years for BMI higher than 30 compared with 1.15 years for BMI 20–29 (χ^2^=19.4, *P*<.001).

**CONCLUSION:**

Disparities exist in the time to endometriosis diagnosis after pelvic and perineal pain diagnosis. Obesity, older age, Black race, and Hispanic ethnicity are associated with longer times from a pelvic pain diagnosis to an endometriosis diagnosis.

## Abstract 2

Older age, obesity, Black race, and Hispanic ethnicity are associated with longer time to endometriosis diagnosis after presentation with pelvic and perineal pain.

Endometriosis is an estrogen-driven inflammatory condition characterized by the presence of extrauterine endometrial glands and stroma.^1–3^ It affects approximately 10% of women of reproductive age with a peak incidence in women aged 25–35 years.^1,4,5^ A wide range of signs and symptoms are associated with endometriosis, with the most common being pelvic pain and infertility.^6,7^ Over the past several decades, understanding of the molecular mechanisms underlying endometriosis and diagnostic and treatment approaches has greatly improved. However, delays in the diagnosis and management of endometriosis persist. With more than 176 million individuals affected by endometriosis worldwide, recognizing and addressing disparities in diagnosis and management is vital to promoting adequate and equitable care.^8^

Despite the significant effect of endometriosis on quality of life, diagnostic delay remains a prevalent issue.^9^ Recent studies have reported varying lengths of diagnostic delays ranging from approximately 4 to 8 years.^10–13^ Data from the All-Party Parliamentary Group in the United Kingdom reveal that patients have upward of 10 visits with their primary care clinician after the onset of symptoms until official diagnosis.^11^ Several contributors to this diagnostic delay have been suggested, including a lack of high-quality questionnaires to assess patients’ risks for endometriosis, limited use of imaging modalities such as ultrasonography in the diagnostic process, limited association between symptoms and stage of disease, lack of sensitive and specific noninvasive biomarkers, and clinician dismissal of symptoms.^9,12,14,15^ However, the extent to which social determinants of health may contribute to diagnostic delay requires further research.

Previous studies have highlighted disparities in the management of gynecologic care overall. For instance, Black individuals, Indigenous individuals, and people of color are less likely to receive minimally invasive surgeries for procedures such as hysterectomies and myomectomies, and they face a higher risk of perioperative complications.^16,17^ Additional factors, including age and body habitus, are known to influence the prevalence of endometriosis. Because the highest prevalence is observed in women aged 25 to 35 years, it is likely that individuals with endometriosis outside this age range may face longer-than-expected delays in diagnosis. Two seminal studies have established an inverse relationship between body size and the risk of endometriosis.^18,19^ Given this established association by health care practitioners or a potential bias against performing laparoscopy for diagnosis in patients with higher body mass index (BMI, calculated as weight in kilograms divided by height in meters squared), it is also likely that individuals with a higher BMI may face a longer time to endometriosis diagnosis.

The primary objective of this study was to describe the associations between age, race, ethnicity, and BMI ranges and the length of time from presentation with endometriosis symptoms to time of diagnosis. We hypothesized that there would be an increase in time to endometriosis diagnosis in individuals of underrepresented populations in terms of race, age, and BMI.

## METHODS

We used the TriNetX research network to conduct this retrospective study, accessing the database in September and October 2024. We used the U.S. Collaborative Network within the TriNetX platform to establish our patient cohorts. This network consists of deidentified electronic health record data from more than 100 million patients from 67 U.S. health care organizations. Health care organizations join the network to receive access to the analytics, deidentified data, and opportunities for sponsored clinical trials of the platform.^20^ Data are deidentified per the Health Insurance Portability and Accountability Act (HIPAA) criteria, Section §164.514(a) of the HIPAA Privacy Rule. TriNetX has an exemption from the Case Western Reserve University IRB because of the data being deidentified and complying with HIPAA.^20^ Thus, this study was IRB exempt. The STROBE (Strengthening the Reporting of Observational Studies in Epidemiology) guidelines were followed for the completion of this study.

**Fig. 1.**
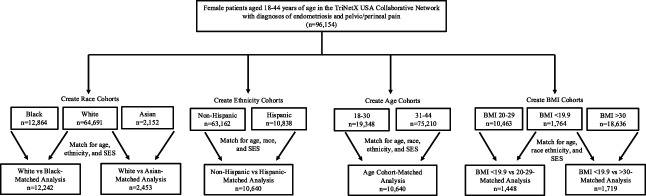
Flow diagram of study design. The age matched for was age at pelvic pain diagnosis (matched using Z codes Z55–65). SES, socioeconomic status; BMI, body mass index.

The total study population consisted of 18- to 44-year-old female patients who received care in the participating U.S. health care systems. The study was designed by querying International Classification of Diseases, Tenth Revision (ICD-10) codes of interest. Of note, ICD-10 codes replaced International Classification of Diseases, Ninth Revision codes in October 2015, so the study population ultimately includes patients since the adoption of ICD-10 coding. All patients included in the study had diagnoses of both endometriosis (ICD-10 N80) and pelvic–perineal pain (ICD-10 R10.2). An additional cohort was created of patients with diagnoses of dysmenorrhea (ICD-10 N94.6) and endometriosis. Cohorts of patients separated by race, ethnicity, age, and BMI cohorts were created a priori. All of these cohorts were created individually, meaning that a patient could be a member of multiple cohorts (eg, an age, race, and BMI cohort) if they had ICD-10–coded information that placed them in the cohort. Race and ethnicity were included in our study because of the previously documented disparities of gynecologic care that have been associated with these patient demographics.^21^ Age cohorts were separated into 18–30 years and 31–44 years. The BMI cohorts were categorized as lower than 19.9 (ICD-10 Z68.1), 20–29 (ICD-10 Z68.2), and higher than 30 (ICD-10 Z68.3, Z68.4). A flow diagram of the study design is shown in Figure 1.

*Imbroane. Disparities in Time to Endometriosis Diagnosis. O&G Open 2025*.

Patients were matched 1:1 for race, ethnicity, and age at pelvic pain diagnosis with the built-in TriNetX propensity score matching function. Propensity score 1:1 matching used a greedy nearest-neighbor approach with a caliper distance of 0.1 pooled SD of the logit of the propensity score. This methodology matches cohorts according to their likelihood of being similar in stepwise fashion by always choosing the most optimal match in the moment. Any characteristic with a standardized mean difference between cohorts lower than 0.1 is considered well matched.^22^ To adjust for socioeconomic status, we also matched on the basis of ICD-10 codes Z55–65, which include codes such as problems related to employment and unemployment and problems related to housing and economic circumstances. We assessed the median time to endometriosis diagnosis after an initial diagnosis of pelvic and perineal pain or dysmenorrhea using Kaplan–Meier analysis and the log-rank test. All instances of pelvic and perineal pain were included, and there was no set time of when this presentation had to occur before the endometriosis diagnosis. Of note, the diagnosis is based on ICD-10 code N80, and we are unable to elucidate how the diagnosis was made in TriNetX (eg, laparoscopy vs imaging studies). Median time to endometriosis diagnosis was compared for the White and Black, White and Asian, Hispanic and non-Hispanic, BMI lower than 19.9 and BMI 20–29, BMI lower than 19.9 and higher than 30, and age 18–30 years and 31–44 years cohorts. Only two-group comparisons can be conducted within the TriNetX platform, so a comparison of all groups within a cohort was unable to be completed at a single time. The final analysis in our initial study design was the same analyses using an initial diagnosis of dysmenorrhea instead of pelvic pain to assess whether similar results were seen for another possible presenting symptom of endometriosis. Statistical analyses were conducted in the TriNetX platform with R 4.0.2, Survival package 3.2-3. A value of *P<*.05 was considered significant.

## RESULTS

**Fig. 2.**
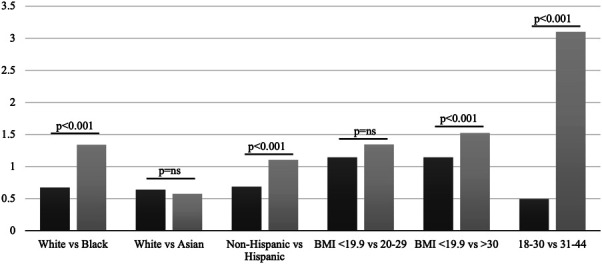
Median years from pelvic pain presentation to endometriosis diagnosis shown by race and ethnicity, body mass index (BMI) range, and age. NS, not significant.

Overall, there were 96,154 patients aged 18–44 years with diagnoses of both pelvic–perineal pain and endometriosis. On post hoc analysis, 8.2% of patients with both of these diagnoses were found to have ICD-10 codes Z55–65. Baseline characteristics of the study population are shown in Table 1. After matching, there were 13,662 patients in each of the age cohorts, 12,242 patients in each of the Black and White cohorts, 2,453 patients in each of the Asian and White cohorts, 10,640 in each of the Hispanic and non-Hispanic cohorts, 1,448 in each of the BMI lower than 19.9 and 20–29 cohorts, and 1,719 patients in each of the BMI lower than 19.9 and higher than 30 cohorts. The median time to endometriosis diagnosis was longer for patients aged 31–44 years (3.10 years) than patients aged 18–30 years (0.49 years, χ^2^=3,503, *P*<.001). Median time to diagnosis was also longer for Black patients (1.34 years) compared with White patients (0.67 years, χ^2^=128, *P*<.001). There was no significant difference in the median time to diagnosis when Asian and White patients were compared: 0.58 years for Asian patients and 0.64 years for White patients (χ^2^=0.68, *P*=.41). In a comparison of Hispanic and non-Hispanic patients, the median time to endometriosis diagnosis was 1.11 years for Hispanic patients and 0.68 years for non-Hispanic patients (χ^2^=27, *P*<.001). There was no significant difference in the time to diagnosis between patients with BMI lower than 19.9 (1.15 years) and those with BMI 20–29 (1.35 years, χ^2^=2.8, *P*=.10). However, patients with BMI higher than 30 had a significantly longer median time to endometriosis diagnosis (1.52 years) compared with patients with BMI lower than 19.9 (1.15 years, χ^2^=19.4, *P*<.001). All analyses are shown in Figure 2.

**Table 1.** Baseline Characteristics of the Study Population: Female Patients Aged 18–44 Years With Diagnoses of Pelvic and Perineal Pain and Endometriosis (N=96,154)

	
Characteristic	Value
Age (y)	36.0±6.0
Race	
Asian	2,317 (2.4)
American Indian/Alaska Native	404 (0.4)
Black or African American	13,692 (14.2)
Native Hawaiian or other Pacific Islander	279 (0.3)
White	66,539 (69.2)
Additional races	4,721 (4.9)
Unknown race	8,202 (8.5)
Ethnicity	
Hispanic	12,135 (12.6)
Not Hispanic	74,221 (77.2)
Unknown	9,798 (10.2)
BMI (n=30,863)*	
Lower than 19.9	1,764 (5.7)
20–29	10,463 (33.9)
Higher than 30	18,636 (60.4)

BMI, body mass index.

Data are mean±SD or n (section of column %).

* Number of patients with BMI recorded.

*Imbroane. Disparities in Time to Endometriosis Diagnosis. O&G Open 2025*.

Analysis was also completed for patients with a preceding diagnosis of dysmenorrhea to corroborate the disparities in time to endometriosis diagnosis after presentation for pelvic and perineal pain. Of note, the starting sample size for this secondary analysis was smaller at 53,567 patients compared with more than 90,000 patients with perineal and pelvic pain. Once again, older, Black, and Hispanic patients had significantly longer times to endometriosis diagnosis. No difference was seen in either BMI cohort analysis; however, sample size was limited to around 1,000 patients for these analyses. Results of this analysis are included Appendix 1, available online at http://links.lww.com/AOG/E56.

## DISCUSSION

Our retrospective study found that disparities do exist in the time to endometriosis diagnosis after pelvic and perineal pain diagnosis based on certain patient characteristics. Patients with obesity and those who are older, Black, and Hispanic have longer median times to endometriosis diagnosis, with the longest delays in endometriosis diagnosis being seen in older patients and Black patients.

The findings of our study should be interpreted in the context of the previously established 4- to 8-year diagnostic delay for endometriosis, specifically 4.4 years in the United States as of 2017.^10–13^ The 2017 study was completed in 2012 and used a cross-sectional survey of patients with an official diagnosis of endometriosis through a method such as laparoscopy or with a physicians' suspicion of endometriosis within the previous 10 years.^10^ A possible explanation for this incongruence is the difference between the time to diagnosis and the time from consultation to diagnosis in these patient-reported data. The time from consultation to diagnosis observed in this study (an average of 12.5 months for patients aged younger than 18 years and 34.5 months for patients aged 40–49 years) shows similarities to our average time of 3.10 years for diagnosis in the 31- to 44-year age cohort in our study. Thus, patients are likely facing delays in diagnosis that are ultimately longer than what our study is showing, reflecting the inability of our study to capture how long patients are experiencing pelvic pain before it is recorded in their medical record.

Our study documented an increased time to endometriosis diagnosis in patients from historically disadvantaged backgrounds. There is a historical misconception that endometriosis is rare in Black women, which has contributed to race-based inequities in care.^23^ Although some research suggests a genetic predisposition to endometriosis, these studies often overlook the effect of varying time to diagnosis across races, which complicates the understanding of prevalence.^24,25^ One study found that age younger than 18 years, Black race, and presenting with symptoms to specialties other than obstetrics and gynecology were significantly associated with increased time to diagnosis; other socioeconomic factors had less of an influence.^10^ However, this study was limited by a smaller sample size and a survey design that required asking patients to recall symptoms and events from up to 10 years in the past. Because of the small number of studies exploring this issue and limitations in study design, our understanding of the social determinants of health as they relate to disparities in time to endometriosis diagnosis needs to be explored further.

Regarding the differences we observed in time to diagnosis by age, one previous study found that women younger than age 18 years experienced a significantly longer time to diagnosis compared with patients in other age groups.^10^ This aligns with reports indicating that adolescents often present with atypical endometriosis symptoms.^26^ However, that same study found a longer time from first consultation, compared with symptom presentation, to final diagnosis of endometriosis in a cohort of patients aged 40–49 years; these data are in line with our findings. Ultimately, further research is needed to explore the reasons why the older cohort of patients in this study are facing the most significant delay in diagnosis observed in our study. Potentially, these patients could have had symptoms controlled with medical management or had milder symptoms, leading health care professionals to delay making a definitive diagnosis. Another possible explanation for the delay in diagnosis in the older cohort is a lack of continuity of care. If this cohort is not receiving consistent care, it could take longer for them to obtain a diagnosis. These potential explanations and others we have yet to hypothesize need to be further investigated with research methodology that can look at individual patient experiences with the health care system leading up to an endometriosis diagnosis.

Finally, we observed that patients with higher BMI faced delays in diagnosis compared with an underweight cohort. This was a result we expected given that the prevalence of endometriosis is higher in patients with a lower BMI.^18,19^ Furthermore, one study has identified delays in diagnosis for patients with obesity compared with those with BMIs in the overweight, normal weight, or underweight range.^27^ However, this study was limited by a small sample size. It is also known that patients with obesity face significant stigma within the health care system.^28^ Therefore, both endometriosis being a less suspected diagnosis in patients with higher BMI and the stigma that these patients face may be reasons for the delay in diagnosis experienced by this patient population. Further studies comparing patients with BMI ranges in categories such as lower than 18.5, 18.5–24.9, 25–29.9, and higher than 30, and patients with varying classes of obesity should also be performed.

Ultimately, given the disparities in time to endometriosis diagnosis observed in this study, clinicians need to be mindful that patients without traditional endometriosis risk factors (lower BMI, younger age) can still develop the condition. In addition, standardized algorithms for pelvic pain or dysmenorrhea should be used to address the implicit bias among health care professionals that may be leading to Black and Hispanic patients facing longer wait times to receive an endometriosis diagnosis. Other areas for improvement in clinical practice include better screening for endometriosis at primary care visits, providing patients with educational materials explaining the symptoms of endometriosis, and reducing the stigma of patients discussing symptoms related to menstruation with their health care practitioner by ensuring that it is a standard discussion at annual primary care and gynecology visits.

Future studies are needed to examine the experience of these patients in the health care system from pelvic pain presentation to finally receiving their diagnosis of endometriosis to see where these diagnostic delays are occurring. There is also a need to evaluate structural and systemic factors that may be contributing to patients' different experiences in the health care system. For instance, delays related to insurance coverage for visits to a gynecologist or diagnostic testing and barriers related to being able to attend appointments could be factors contributing to the disparities observed in this study. From our study, it is unknown whether these patients facing numerous visits before a clinician chooses to proceed with a diagnostic procedure, experiencing longer wait times for laparoscopy or imaging studies, undergoing several lines of medical management, or other factors prolong the wait.

The main strength of this study was the large sample size conferred by the use of the TriNetX database. However, the study is not without its limitations. The reliance on ICD-10 coding is a weakness of the study given that coding practices may vary among clinicians, and we are unable to assess the validity of the coding. Although we were able to conduct matched analyses, we were not able to perform adjusted analyses or to compare more than two cohorts with each analysis. We were also unable to assess how the diagnosis of endometriosis was made by clinicians, only that it was coded. Thus, some patients may be undergoing laparoscopy, others may have imaging studies, and some may even be diagnosed on the basis of clinical suspicion from the clinician. Another limitation is related to laparoscopy being the gold standard to diagnose endometriosis, with patients with lower socioeconomic status possibly having increased difficulty in accessing an invasive procedure or even consistent follow-up or taking time away from work or other responsibilities, thereby yielding an inherently longer time to diagnosis. Patients with higher BMI may also be less likely to be offered this procedure. To try to account for this limitation, we matched for ICD-10 codes Z55–65 as a proxy for socioeconomic status. Finally, we were unable to ascertain whether patients were being controlled on medical management under presumed yet not formally diagnosed endometriosis by health care professionals, which could be contributing to a delay in ICD-10–coded diagnosis on its own.

Our study provides evidence that obesity, older age, Black race, and Hispanic ethnicity are associated with longer time to endometriosis diagnosis. Alterations to clinical practice to narrow the gap between time to endometriosis diagnosis among these patient populations should be considered.
